# Using Smart Phone Sensors to Detect Transportation Modes

**DOI:** 10.3390/s141120843

**Published:** 2014-11-04

**Authors:** Hao Xia, Yanyou Qiao, Jun Jian, Yuanfei Chang

**Affiliations:** The Institute of Remote Sensing and Digital Earth, No.20 Datun Road, Chaoyang District, Beijing 100101, China; E-Mails: qiaoyy@radi.ac.cn (Y.Q.); jianjun@radi.ac.cn (J.J.); changyf@radi.ac.cn (Y.C.)

**Keywords:** transportation mode classification, built-in sensor, smart phone, trajectory

## Abstract

The proliferation of mobile smart devices has led to a rapid increase of location-based services, many of which are amassing large datasets of user trajectory information. Unfortunately, current trajectory information is not yet sufficiently rich to support classification of user transportation modes. In this paper, we propose a method that employs both the Global Positioning System and accelerometer data from smart devices to classify user outdoor transportation modes. The classified modes include walking, bicycling, and motorized transport, in addition to the motionless (stationary) state, for which we provide new depth analysis. In our classification, stationary mode has two sub-modes: stay (remaining in the same place for a prolonged time period; e.g., in a parked vehicle) and wait (remaining at a location for a short period; e.g., waiting at a red traffic light). These two sub-modes present different semantics for data mining applications. We use support vector machines with parameters that are optimized for pattern recognition. In addition, we employ ant colony optimization to reduce the dimension of features and analyze their relative importance. The resulting classification system achieves an accuracy rate of 96.31% when applied to a dataset obtained from 18 mobile users.

## 1. Introduction

In recent years, Location-Based Service (LBS) and Mobile Social Networks (MSNs) have grown rapidly. Typically, a user accesses these services through a mobile smart device capable of providing user location via the Global Positioning System (GPS), cell tower, Wireless Fidelity (WiFi), Radio Frequency Identification (RFID), Bluetooth, or some combination of these mobile communication components. User trajectories can then be formed from location points according to certain rules. By analyzing the variety of spatial and temporal trajectory data from users, new insights can be gained in fields such as behavioral psychology, transportation and logistics, urban design, emergency response, and many others.

Although common location-based tracking can adequately capture user trajectories, it is limited in supporting new and more refined applications of mobile user data mining. For example, in analyzing “hot paths” (*i.e.*, paths followed by a large number of user trajectories), we generally assume that such paths demand greater geo-spatial attention from the user in motion [1–3]. However, for the same hot path, traveling in different transportation modes (walking, bicycling, driving/riding, *etc.*) implies very different interpretations. Some scholars have focused on data models that represent moving objects for varied transportation modes and contexts [4–8]. These models serve as a basis for applications in various fields ranging from transportation and logistics to ecology and anthropology [9]; however, large-scale application of such models remains hindered by lack of an efficient and effective means of detecting user transportation modes.

Further complicating the issue of multi-modal trajectories is the fact that most tracking and recording is performed as a background process on mobile devices, such as by updating locations at regular intervals or according to some other stable parameter (e.g., distance). Users cannot be expected to supplement this background process by marking significant start/end points and/or by specifying modes of transportation. To supply this crucial information, a robust and scalable method of classifying segments of user trajectories according to transportation mode is required.

Methods for transportation mode classification and human motion recognition from a variety of perspectives have already been proposed. In [10,11], for example, the authors propose approaches to inferring people's transportation mode based on GPS tracking and knowledge of the underlying transportation network. In [12], accelerometers and gyroscopes are used to analyze driving habits. In [10,13], a combination of GPS and Geographic Information System (GIS) is proposed for identification of transportation modes. The methods presented in [14–19] extract acceleration information within the frequency range of the human gait and thereby implement gait pattern classification. Similarly, in [20], the author extracts gyroscopic data features to classify leg motions. Although instructive, most of these studies focus on highly specific application fields (e.g., medical care) and/or are too dependent on high-precision sensors to enable achievement of more broadly defined aim. While a few studies have proposed techniques for classifying common transportation modes, the classification models used by these methods are not sufficiently refined or robust for wide use. Finally, unlike some of the above studies, we wish to avoid use of a GIS spatial database because it would involve infeasible restrictions.

To date, transportation modes have been generally divided into four classes: stationary, walking, bicycling, and motorized driving/riding. Although these classes support many useful data mining operations, adequate consideration has not been given to the *overlapping* of stationary and other transportation modes. In most studies, intermittent records whose velocity values are zero are simply deleted from analysis unless they can be confidently classified as stationary mode. Nevertheless, these intermittent stops are significant; e.g., being stopped at a traffic light is distinct from sitting in a parked car, just as moving at a high speed is distinct from traveling slowly. Such distinctions are especially important when the attention level of the user is to be assessed.

**Figure 1.**
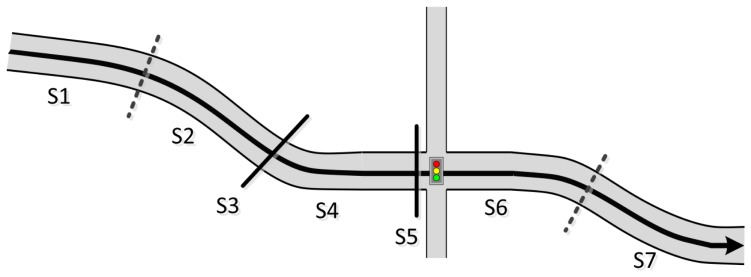
Object moving on a roadway; the trajectory is defined as a series of sections. S1, S2, S4, S6, and S7 are *moving* sections; S3 and S5 are *stationary* sections.

To delineate the differences among these modes, we refine our classification of transportation modes as follows. Stationary mode occurs when the velocity value remains at zero or below a certain threshold for some time span; moving mode is the opposite of stationary mode. As shown in Figure 1, the trajectory is a sequence of alternating moving and stationary sections. Distinguishing these sections within a trajectory is the primary function of the application [9].

As shown in Figure 1, we understand the trajectory to be is a sequence of moving sections alternating with stationary sections. Distinguishing these sections within a trajectory is the primary responsibility function of the application [9].

Moving mode and stationary mode are then subdivided. Moving mode includes sub-modes for walking, bicycling, and motorized driving/riding, while stationary mode is divided into stay and wait sub-modes. Stay mode is defined as remaining in place for a certain period, with neither vibrations nor forward movement (*i.e.*, velocity is close to zero). In wait mode, the speed is again close to zero; however, vibration is detected (e.g., the vehicle has stopped; however, the engine is running, which produces the vibration). Distinguishing these low-speed modes is helpful in many applications. For example, it can foster an understanding of popular areas of waiting and long-term staying, and then provide targeted information to surrounding users. Moreover, the distribution of wait sections can provide information on traffic levels, estimations of additional fuel consumption for congested areas.

Based on these refined transportation modes, we propose a transportation mode classification method based on the common array of built-in sensors provided by smart phones. Specifically, our method combines acceleration with speed obtained by GPS receivers by using optimized Support Vector Machine (SVM) [21] classification to achieve better classification results. In addition, we use Ant Colony Optimization (ACO) [22,23] in our feature extraction to reduce the SVM feature dimensions and determine the key factors for identifying various transportation modes.

The remainder of this paper is organized as follows: in Section 2, we detail the theory and techniques used in the proposed method. In Section 3, we outline the experimental process using the proposed method, and we describe and analyze the results in Section 4. In Section 5, we present our concluding remarks.

## 2. Method

The major steps of transportation mode classification include: collecting data with smart phones, extracting features from the data, training the classifier, and classifying transportation modes using the trained classifier. Before data collection, we must first determine the sensors we need, as well as the parameters such as sampling frequency, time window, *etc.* These works are introduced in Sections 2.1 and 2.2. The feature types we need and methods to obtain these features are described in Section 2.3. Section 2.4 introduces the classifier design. The ACO described in Section 2.5 is not a necessary step of transportation mode classification at each time; however, it can analyze and filter features. ACO is an important optimization tool.

### 2.1. Sensor Selection

Mobile phones typically include GPS for outdoors localization and an Accelerometer Sensor (AS), a Compass Sensor (CS), a Gyroscope Sensor (GS), an Image Sensor (IS), an Ambient Light Sensor (ALS), a Proximity Sensor (PS), Touch Sensors (TS), a Temperature Sensor (TS), a Humidity Sensor (HS) and an Atmospheric Pressure Sensor (APS) [24]. These sensors can provide raw data with high precision and accuracy; moreover, they are particularly useful for monitoring three-dimensional device movement or positioning, or ambient environment changes near the device [25].

When performing transportation mode classification based on sensor input, a single sensor type is not ideal; it typically results in an accuracy drop of 10% to 20% compared to classification using multiple sensor input [26]. Generally, two sensor types are desired: motion sensors (e.g., AS, GS, *etc.*) and position sensors (e.g., GPS receivers). In transportation mode classification, motion sensors provide vibration or oscillation characteristics, while position sensors are directly or indirectly used to obtain speed information. Because most mobile smart devices are equipped with both motion and position sensors, we have chosen to base our classification methods on data from those sensors.

**Figure 2.**
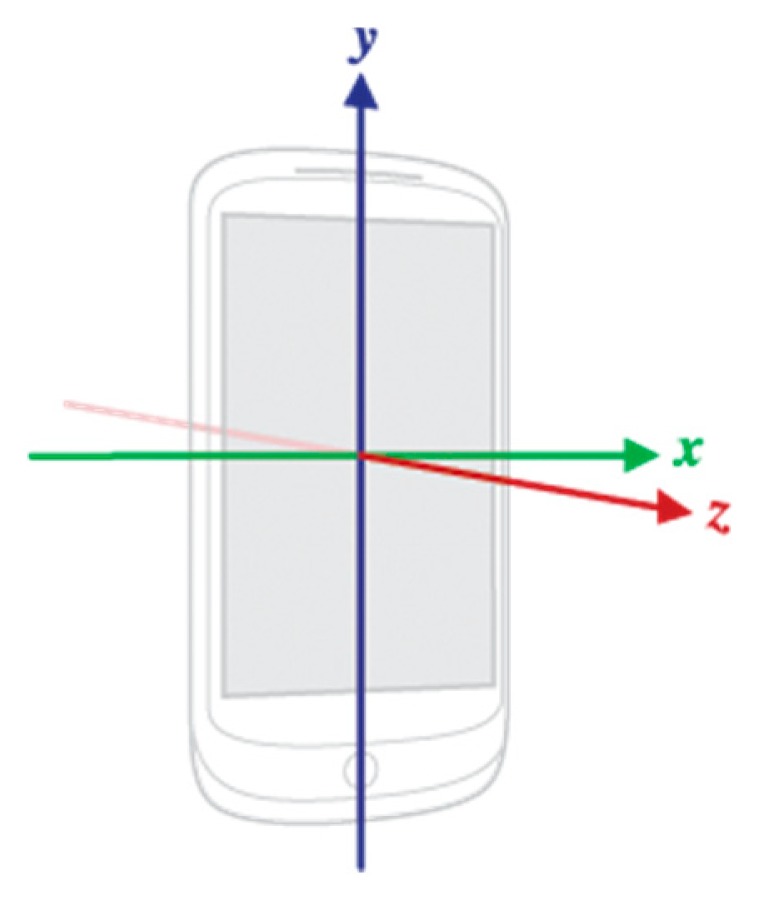
Coordinate system in relation to a mobile smart device.

As previously mentioned, in several related studies [14–19], the accelerometer is used to obtain vibration/oscillation features of users’ bodies or their transportation mode. Although [12] uses both an AS and GS to analyze user driving habits, the primary GS function is rotation feature extraction. Moreover, not every type of mobile device is equipped with a GS. Thus, we limit our motion sensing capacity to what is independently provided by the accelerometer. As shown in Figure 2 (Internet image [27]), the device assumes a standard three-axis coordinate system to express data values.

This coordinate system is defined in relation to the device screen when the device is held in its default orientation. In this orientation, the *x* axis is horizontal and points to the right, the *y* axis is vertical and points up, and the *z* axis points outward from the screen facade. In this system, coordinates behind the screen have negative *z* values.

During some physical activities, a worn or carried smart device may be rotated and reversed relative to the user's body. Thus, we use the magnitude of force vector *A* by combining acceleration measurements from all three axes (*x*, *y*, and *z*) in relation to the device screen as the basis for feature extraction:

(1) $$A=\sqrt{x^{2}+y^{2}+z^{2}}$$

As shown in Table 1, multiple location sensing sources exist for mobile smart devices, including GPS, cell towers, and WiFi. Location sensing based on cell towers and WiFi typically calculate velocity using consecutive location points [26]. On the other hand, GPS-based location sensing can directly determine velocity. Because Bluetooth is typically used in indoor location systems [28,29], we do not consider it.

Given the low accuracy of cell towers and WiFi-based location sensing, neither of which can directly determine velocity, we rely on GPS to determine velocity in this study.

### 2.2. Parameters of Data Collection

#### 2.2.1. Time Window

In this study, we employ a standard 5 s time window. An overly small window size results in classification inaccuracies because the periodicities of certain features within a small time window are not clearly evident. Further, an overly large window size tends to introduce noise from multiple classifiable activities occurring in the same window [26]. The detection of cyclic upright activity characteristic of walking requires at least 3 s to accumulate data [30]. A window size of 5 s ensures that the classifiable data is precise and effective.

#### 2.2.2. Sampling Frequency

We establish a sampling frequency of 50 Hz. The frequency components of body motions are known to be below 20 Hz [30]. In gait recognition research, for example, 99% of the energy of body motion is concentrated in signal components below 15 Hz. As some studies [12] suggest, when an engine is turned on, the vibration frequency of the vehicle seat is 3 to 5 Hz even though the vehicle speed is zero. Therefore, our Nyquist frequency must be higher than 20 Hz. If our sampling frequency is too low, it will produce aliasing and result in a loss of high frequency signal components. If the sampling frequency is too high, the amount of data collected by sensors will be too great, thereby creating a burden on the Central Processing Unit (CPU), memory, and battery resources and sharply increasing the workload for data analysis. Thus, 25 Hz is a sound choice for our Nyquist frequency. Because sampling frequency is double the Nyquist frequency, our sampling frequency is 50 Hz.

#### 2.2.3. Data Elements

We record measurements from the triaxial acceleration sensor and velocity, latitude, and longitude readings from GPS. The timestamp and subject name are recorded.

### 2.3. Feature Types

As mentioned in Section 2.1, in transportation mode classification, the vibration characteristics from acceleration signal and velocity data will be used. We attempt to analyze the vibration characteristics from frequency domain and time domain. Discrete Fast Fourier Transform (DFFT) is the most important discrete transform, used to perform Fourier analysis in many practical applications [31]. We select DFFT features to represent frequency-domain characteristics of acceleration. First proposed by Richman [32,33], Sample Entropy (SampEn) is used extensively for assessing the periodicity of a physiological time-series signal and shows good traits such as data length independence and trouble-free implementation. A SampEn feature is used to reflect the periodicity of acceleration. We use statistical features of velocity.

#### 2.3.1. Discrete Fast Fourier Transform

We use DFFT to extract the acceleration frequency-domain features. In some studies [14–16,19,20], wavelet transform-based methods are used to extract similar features. Ayrulu–Erdem, for example, used the Discrete Wavelet Transform (DWT) and multi-layer feed-forward Artificial Neural Networks (ANNs) to classify leg motions [20]. Nyan selected noise-filtering algorithms to extract feature vectors from a sampling space of different scale wavelet coefficients [16]. Sekine *et al.*, used the energy features of low frequency wavelet coefficients from an acceleration signal in forward and vertical directions for classification [19]. Wang *et al.* [15] used variance with the Root Mean Square (RMS) of two to six layer wavelet coefficients of the acceleration signal as classification features. Preece *et al.* [14] compared time-domain features with wavelet decomposition features. In [26], DFFT was used to extract the features in certain frequencies. The above studies benefitted from known preconditions; namely, the general distribution of feature frequency. Accordingly, they were able to engage specific layers of wavelets or wavelet packet decompositions, or use DFFT to directly select a specific frequency signal. For our purposes, we expect to encounter a wider range of objects and cannot ensure distribution of feature frequency. We use DFFT within the standard time window to directly supply features to our pattern recognition methods. Nevertheless, this approach leads to the familiar problem of feature dimension explosion; therefore, we introduce a method for reducing the dimensions of features in subsequent sections.

#### 2.3.2. Sample Entropy

The greater the value of the SampEn, the more complex is the sequence and the worse is its periodicity. By contrast, the smaller the SampEn value, the better is the periodicity of the sequence. SampEn has been used for characterizing aphasias (e.g., stuttering) [34]; moreover, in [35], it was proposed for intelligent prognosis of battery health. Similarly, the acceleration signal from typical body exercises produces a periodical and non-stationary time series that can be used to express the degree of complexity of SampEn.

#### 2.3.3. Velocity

We choose as features the maximum, minimum, mean, and standard deviation of velocity values from the GPS receiver. The mean shows the concentration of velocity values, while the standard deviation reflects the dispersion of values. Maximum and minimum yield the characteristics of various transportation vehicles. GPS points are often invalid when the phone is significantly shielded or if the user is in an occluded area, such as a tunnel or arcade. To address this issue, invalid GPS points are suppressed based on the GPS accuracy [10,26].

### 2.4. Classification

#### 2.4.1. Mode definitions

When the velocity value of samples is greater than a certain threshold, the user is considered to be in moving mode; otherwise, the user is in stationary mode. This threshold is employed by a specific filter, which is described further below. Moving mode sub-modes are defined in Section 1. As mentioned, stationary mode includes stay and wait sub-modes. For example, a user in a parked vehicle or seated elsewhere is in stay mode, even though some body movements are exerted. A user in wait mode is in a travel pause state (e.g., being stopped at a traffic light) and typically yields certain regular vibrations. By extension, wait mode can be divided into three sub-modes: wait-while-walking, wait-while-biking, and wait-while-motoring.

#### 2.4.2. Classifier

**Figure 3.**
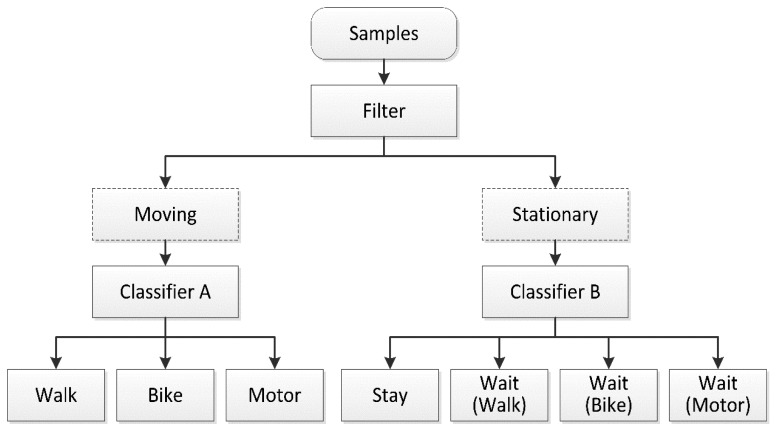
A filter divides samples into stationary and moving modes according to a velocity threshold. Classifiers A and B are SVMs.

As shown in Figure 3, a speed threshold must be set for the filter operation to enable dividing of the samples into moving and stationary modes. The samples in these modes are classified by SVM Classifiers A and B. SVM has shown excellent capabilities in resolving problems involving small samples, nonlinearity, regression, and classification of high-dimensional patterns. Compared to traditional neural networks, the SVM structure is simple and its processes are much faster. Moreover, SVM generalizes better and is easier to implement [36]. Related research using SVM has achieved satisfactory results [37–39].

SVM is designed to classify only two types and must be extended to solve multi-classification problems. Examples of such extensions include one-versus-all (OVA) [40], one-versus-one (OVO) [41], Decision-directed Acyclic Graph (DAG) [42,43], among others. The basic idea behind these extensions is to divide a multi-classification problem into multiple dual-classification problems, and then to normally apply SVM.

In [44], several commonly used multi-classification SVMs (OVA, OVO, DAG, *etc.*) were compared. The results showed that the OVO method outperformed other methods in most circumstances. We have therefore chosen OVO for our multi-classification problem. In this study, the LIBSVM was used [45].

Note that the classification results of SVM will be far from ideal if the input samples are imbalanced. To improve the performance of SVM against imbalanced datasets, we use the Synthetic Minority Over-sampling Technique (SMOTE) [46] to acquire smaller number categories. Furthermore, because penalty parameter *C* and kernel parameter *g* are the key parameters affecting the performance of the SVM classifier, we use a grid search algorithm to optimize these variables. Ten-fold cross validation will be used in optimization.

### 2.5. Feature Analysis and Extraction

We use the improved ACO to filter unimportant features and reduce the input dimensions for SVM. ACO is typically used for combinatorial optimization problems. Based on its success in solving the Traveling Salesman Problem (TSP), it has drawn interest from a number of scholars [47,48]. Because it differs from the use of ACO in solving the TSP, we describe our own use of ACO.

First, we select *num_f_* features from all *N* features to support transportation mode classification. If we consider a feature as a city, *N* cities exist for constructing an undirected complete graph. The graph edges are of equal length. An ant will go through *num_f_* cities between its origin and destination. Ants spend equal time covering the distance between any two cities because all paths between two cities are of equal length. If we use the time spent on the path between two cities as a unit, an ant always starts from its origin city at time *t* = 0 and will reach its destination city at time *t* = *num_f_* - 1.The total number of ants is *m*. Our aim is to find the opposite cities to achieve the best paths. The best path produces the highest classification accuracy. In other words, the best *num_f_* features are chosen to produce the highest classification accuracy.

To communicate with the others, each ant deposits a chemical substance, called a pheromone, on the ground where it walks. This substance evaporates over time, thereby decreasing the intensity of the pheromone. This process is used to avoid being trapped in a local extreme value [49]. When choosing between two paths, ants prefer probability to choose a path with more pheromones (higher probability); in other words, more ants pass it on average [49]. Unlike the classic TSP algorithm, we focus on the attractiveness of a city (feature) to ants, not a path, and give no consideration to previous cities through which the ants have traveled. *τ_j_*(*t*) is the amount of pheromone trail in city *j* at time *t*. At time *t* = 0, the pheromone trail of each city is an equal positive constant. In short, *τ_j_*(0) is positive constant *C*. ρ is the pheromone trail evaporation rate. Δ*τ_j_* is the pheromone level increment:

(2) $$\tau_{j}\left(t+1\right)=\left(1-\rho \right)\tau_{j}\left(t\right)+\Delta \tau_{j}$$

From the above, we set:

(3) $$\Delta \tau_{j}=\frac{r_{\text{best}}}{{\text{num}}_{f}}$$

as the method for updating pheromone trails. *r_best_* is the best classification result for the current iteration. The better the classification result, the more pheromones that city *j* acquires. The greater the number of available cities, the fewer pheromones each city receives.

In TSP, *η_ij_* is the *a priori* available heuristic information from city *i* to *j*, which is inspired by the shortest path searching behavior of various ant species [48]. This is typically 1/*d_ij_*, where *d_ij_* is the distance between city *i* and *j*. Because all cities construct an undirected complete graph with equal edge lengths, the order of cities and distances between them are ignored. Therefore, we use *η_j_* as heuristic information for city *j*. We construct the expression *η_j_* = F− *score_j_*. = *F*− *score_j_*. [50,51] is a simple measurement used to evaluate the discrimination ability of a feature. Equation (4) defines the *F*-score of the *j*th feature. The numerator specifies the discrimination among the categories of the target variable; the denominator indicates the discrimination within each category. A larger *F*-score corresponds to a greater likelihood that this feature is discriminative [50,51]:

(4) $$F–{\text{score}}_{j}=\frac{\sum_{c=1}^{v}{\left({\bar{x}}_{j}^{c}-{\bar{x}}_{j}\right)}^{2}}{\sum_{c=1}^{v}\left\{\frac{1}{N_{j}^{c}-1}\sum_{k=1}^{N_{j}^{c}}{\left(x_{jk}^{c}-{\bar{x}}_{j}^{c}\right)}^{2}\right\}},j\in \left\{1,2,\cdots ,N_{F}\right\}$$

where *υ* is the number of categories of the target variable; *N_F_* is the number of features; $N_{j}^{c}$ is the number of samples of the *j*th feature with categorical value *c*, *c*∈ {1,2,⋯,*υ*}, *j*∈ {1,2,⋯,*N_F_*}; $x_{jk}^{c}$ is the *k*th training sample for the *j*th feature with categorical value *c*, *k*∈ {1,2,⋯, $N_{j}^{c}$}; $\bar{x_{j}}$ is the mean of the *j*th feature; and $\bar{x_{j}^{c}}$ is the mean of the *j*th feature with categorical value *c*.

$p_{j}^{k}(t)$ is the probability of ant *k* selecting *j* as the next city at time *t*. *α* indicates the degree of importance of pheromones. *β* indicates the degree of importance of heuristic information. *tabu_k_* is the set of cities through which ant *k* has passed. The cities in *tabu_k_* will not be selected again in an iteration. The relationship between the parameters is expressed as:

(5) $$p_{j}^{k}\left(t\right)=\{\begin{array}{c} \frac{\tau_{j}^{\alpha }\left(t\right)\eta_{j}^{\beta }}{\sum^{\tau_{\gamma }^{\alpha }}\left(t\right)\eta_{\gamma }^{\beta }},\gamma ,j\notin {\text{tabu}}_{k}\\ 0,j\in {\text{tabu}}_{k} \end{array}$$

At this point, we can implement ACO after the feature set is input into the ACO-SVM method.

**Figure 4.**
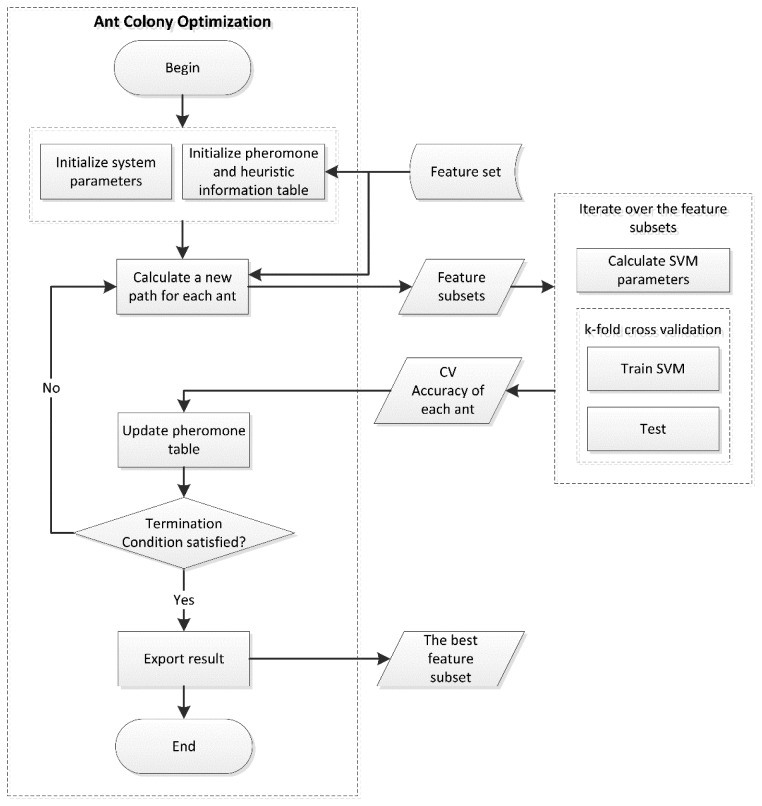
System architecture for proposed ACO-based feature extraction.

To precisely establish an ACO-based optimization method that can filter features and reduce the input dimensions for SVM, the following main steps must be performed, which are illustrated in Figure 4:

Step 1. Initialize parameters and variables of the proposed algorithm: initialize the total number of ants, the time of iterations, the pheromone of each feature, *α*, *β*, the number of selected features *num_f_*, and so on. Then, calculate the *F-*score of each feature as heuristic information using Equation (4).
Step 2. In the first iteration, *num_f_* features are randomly assigned to each ant as the input of SVM. Equations (2) and (3) are then used to update *τ*.
Step 3. Use Equation (5) to calculate the probability of ant *k* selecting cities. To avoid a local over-optimal solution, the roulette wheel selection algorithm is used for the selection strategy. After moving the selected city to *tabu_k_*, we calculate the probabilities of the rest. The ant selects the next city not in *tabu_k_* using roulette wheel selection until *num_f_* cities are chosen. This is our feature subset.
Step 4. Iterate over the feature subsets of all ants, and perform classification using SVM. In each iteration, new SVM parameters are selected using the grid search algorithm. The mean of the results of ten-fold cross validation will be outputted. After updating *τ_j_* using Equations (2) and (3), *tabu_k_* will be emptied and the next iteration will begin.
Step 5. When reaching the specified number of iterations, we obtain the best subset with the highest classification accuracy.

## 3. Experiments

In this section, the experimental details are presented, including the hardware and applications used, subjects, processes of data collection, and processing. Then, we introduce the dataset we obtained and attempt to analyze it. At the end of this section, the ACO results are shown by listing the most important features for classification.

### 3.1. Hardware

Our experiments made exclusive use of HTC J Butterfly smartphones. These devices have a quad-core processor with 2 GB of Random Access Memory (RAM). They run the Android 4.1.2 operating system and employ the BMA250 built-in acceleration sensor. As outlined in Table 2, the BMA250 is a triaxial, low-g acceleration sensor with digital output [52].

Our data processing software was run on a Personal Computer (PC) with a 2.30 GHz Intel Core i5-2410M CPU and 8 GB of RAM.

### 3.2. Software

The software used in this work includes two parts: an application on a smart phone to collect data and series data processing tools on a PC.

#### 3.2.1. Application

**Figure 5.**
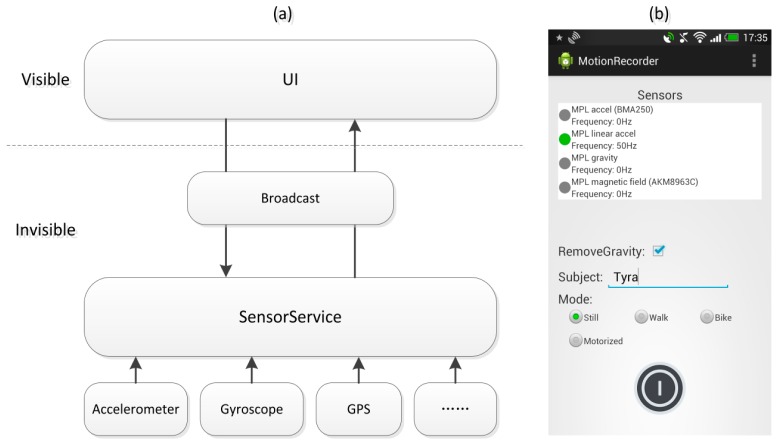
Data collection application: (**a**) structure; and (**b**) screenshot.

A Mobile Sensing System (MSS) requires a user level Application running on the phone for reading an internal phone's sensor [24]. To more accurately and efficiently collect transportation mode data, we developed a special data collection application, which is illustrated in Figure 5.

Our application is comprised of a sensor service and user interface. The sensor service is an independent process that collects data from location and motion sensors and stores it in the Secure Data (SD) card of the smart phone. The sensor service is controlled through the user interface, which communicates with the sensor service through the Broadcast of Android system. This reduces the impact of the user interface on the data collection process and prevents the user interface thread from being blocked by the high-frequency sensor data collection of the sensor service.

#### 3.2.2. Data Processing Tools

We used MATLAB R2013b as the platform to implement this set of tools, including DFFT, ACO, SampEn, and so on. The classifier was based on LIBSVM [45].

### 3.3. Data Collection

Eighteen subjects participated in our experiment (10 males and eight females, ages 23 to 52, with heights ranging from 160 to 186 cm and weights ranging from 40.5 to 96 kg). The smartphones were situated in participants’ jacket pockets (near the waist) during data collection to prevent arm motions from influencing data collection. Participants were tasked to walk, bicycle, motor, and stand. For our purposes, walking speed was treated as a natural speed with no fixed limit. Participants rode common commuter bicycles, and they traveled in buses or cars in urban areas to simulate daily commuter travel. Records included transportation mode flags set by the subjects to verify classification results.

The frequency of acceleration sampling was 50 Hz, and the GPS data collection frequency was 1 Hz. Each subject collected approximately 12 min of data for each transportation mode. Each trajectory data was divided into segments of 5 s of continuous data (our chosen time window) for the same collector and mode. Our dataset was comprised of 16,109 segments; the segment numbers in various modes are showed in Table 3.

### 3.4. Data Processing and Analysis

As mentioned in Section 2.1, in transportation mode classification, we need two aspects of features: velocity and vibration. In this section, further details of the dataset are described. We analyzed the velocity from the statistical distribution, and vibration from the time-domain and frequency-domain characteristics of acceleration.

#### 3.4.1. Velocity Analysis

**Figure 6.**
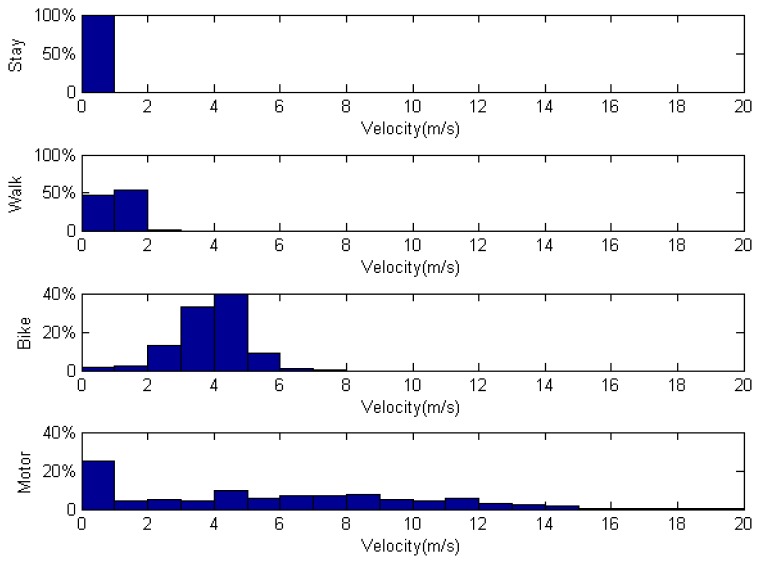
Distribution of velocity among different transportation modes.

The distribution of velocity among different transportation modes is outlined in Figure 6. In stay mode, almost all recorded velocities were 0. Participant walking speeds ranged from 0 to 3 m/s. Velocities for biking and motoring showed a broader distribution.

As shown in Figure 6, the percentage of participant records with a velocity near 0 when traveling in a motor vehicle was greater than 20%. To reflect the general situation of daily vehicular commuters, data collection was performed in urban areas of Beijing, where traffic is tightly managed by traffic signals and there are often gridlocks during rush hour.

An overview of collected data prior to processing is additionally provided in Figure 6. Typically, wait mode is a short transition state and is difficult to accurately record. For example, when traveling in a bus, drivers frequently come to a full stop for more than 5 s. In such cases, it is almost impossible to mark the frequent changes in transportation mode because it cannot be known when the drivers will stop or continue.

Thus, we used a threshold very close to zero (0.05 m/s) to determine whether one is in wait mode. This filter divided our dataset into moving and stationary segments. If the initial mode of a segment was walk mode, but the average velocity fell below the threshold, we sub-classified the segment under the wait-while-walking mode. The features were then extracted from these segments. The segment numbers after filtering are showed in Table 4.

#### 3.4.2. Acceleration Analysis

We then analyzed the time-domain and frequency-domain characteristics of sampled acceleration. To make the frequency-domain characteristics more apparent, we filtered the spectrum (b) of the Direct Current (DC) component (frequency is zero).

**Figure 7.**
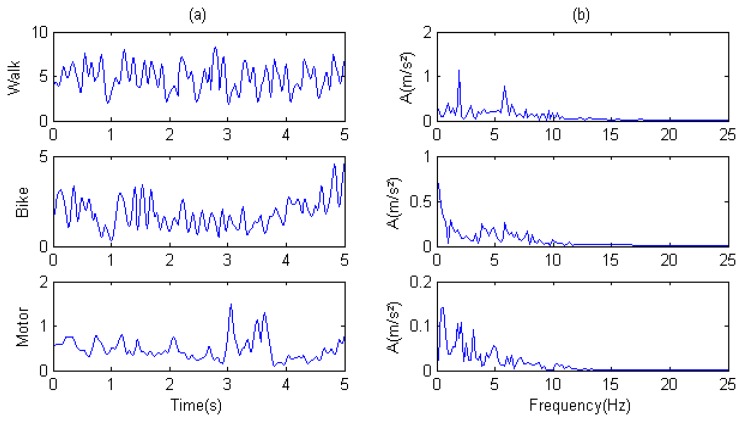
Analysis of mobility sub-modes: walking, bicycling, and motoring: (**a**) acceleration time-domain analysis; and (**b**) acceleration frequency-domain analysis.

Figure 7 shows the acceleration frequencies of three mobility sub-modes distributed across a 0–15 Hz band. Walking shows the most obvious periodicity with two distinct peaks on the spectrum at 6 Hz and 2 Hz. By contrast, motoring shows no obvious periodicity. The samples in three modes are distinguished by amplitudes.

**Figure 8.**
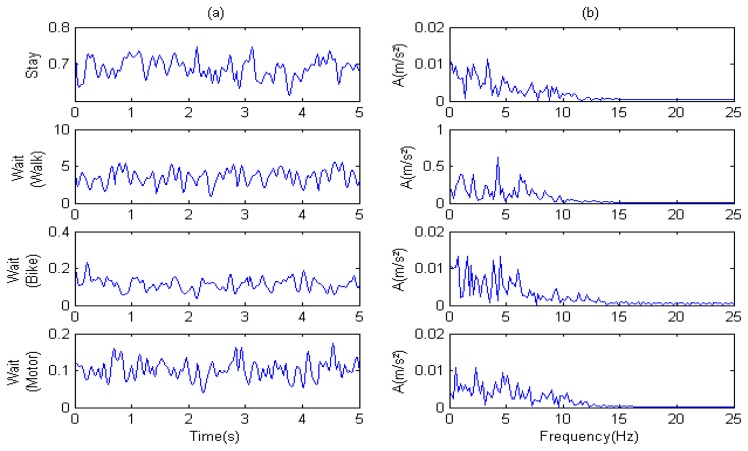
Analysis of stationary sub-modes. Stay and wait modes (including wait-while-walking, wait-while-bicycling, and wait-while-motoring): (**a**) time-domain analysis of acceleration; and (**b**) acceleration frequency-domain analysis.

As shown in Figure 8, all major components of acceleration were between 0 and 15 Hz. The amplitude was different for each mode and showed no significant periodicity.

According to the analysis above, it is difficult to extract features for classifying transportation modes using the methods based on wavelets and wavelet packets. For example, from the spectrum analysis, we cannot directly determine which band is best suited to making distinctions. Therefore, we used the result of DFFT as features for input into SVM after their dimensions were reduced according to previously mentioned methods.

From the perspective of the time domain, the characteristic periodicity of modes was only obvious in walk mode. To explore periodicity types in different modes, we used the values of SampEn mentioned in Section 2.3. The values became input features for SVM, along with the features from velocity and DFFT.

### 3.5. Feature Extraction

We used ACO for filtering to reduce the SVM input sample dimensions. The ant colony size was given by $\sqrt{n}\sim n/2$. In our experiment, the number of features was 134; therefore, the colony size was set to 12. The importance of both pheromone (*α*) and heuristic information (*β*) was set to 0.5. The pheromone evaporation coefficient was 0.4. We randomly selected 100 samples from each mode. The number of iterations was 150.

The Radial Basis Function (RBF) kernel is a popular kernel function used in SVM classification [53]. We use RBF as the kernel function of the SVM in this work [45]. We used a grid search algorithm to optimize our SVM parameters (*C*, *g*). The range of *C* was 2^−8^–2^32^; and the range of *g* was 2^−16^–2^16^. The step of both *C* and *g* was 2^0.8^. We used the average accuracy of ten-fold cross-validation to compare parameters. In each iteration, new SVM parameters were selected because the SVM input sample dimensions changed.

In moving mode, when only seven features were used, accuracy approached the maximum. Between 20 and 80 features produced similar and relatively stable results. Beyond 80, the accuracy began to degrade. In stationary mode, when we selected 1 to 20 features, the accuracy rapidly increased. This was followed by a slowly rising trend approaching a maximum at 117 features, where the accuracy was highest. As a reasonable compromise between recognition accuracy and quantity of features, we selected seven and 30 features with the highest pheromone trails to detect moving and stationary modes, respectively.

## 4. Results and Discussion

In this section, we provide the results of our classification and evaluate the model in terms of classification performance with confusion matrices. In addition, we analyze the importance of each feature in the classification process. An example is demonstrated at the end of the section.

### 4.1. Classification Results

#### 4.1.1. Accuracy and Computing Time

Accuracy is a measure of the overall correctness of the model and is calculated as the sum of correct classifications divided by the total number of classifications. When we selected seven and 30 features, the 10 results of ten-fold cross validation were averaged to produce a single estimation, yielding a total classification accuracy of 96.31%. When we selected all 134 features, the accuracy was 98.54%.

The average computing time of the ten-fold cross validation is shown in Table 5. Although more time intensive, 134 features provided a higher accuracy than 37 features. Otherwise, fewer features would have required less storage and would have more easily transmitted by the network if required. Because there was no profound difference between these two accuracies, we tended to select seven and 30 features.

#### 4.1.2. Confusion Matrices

A confusion matrix [54] contains information about actual and predicted classifications performed by a classification system. Tables 6 and 7 show the averages of the confusion matrices from ten-fold cross validation using seven and 30 features. The proportion of correctly classified samples for each transportation mode can be read on the diagonal (boldface numbers).

Although the overall classification accuracy was satisfactory, the proportions of correctly classified samples for wait-while-biking (0.9227) and wait-while-motoring (0.7773) were relatively low. We determined that these two modes were easily confused. Moreover, some of the sections in wait-while-motoring were classified as stay mode. Some of these results were due to data being recorded by subjects who did not promptly update their changes in transportation mode. Further, some drivers opted to turn their engines off or allowed them to stall while waiting at long red lights, producing vibratory characteristics strongly associated with stay mode.

### 4.2. Critical Features

**Figure 9.**
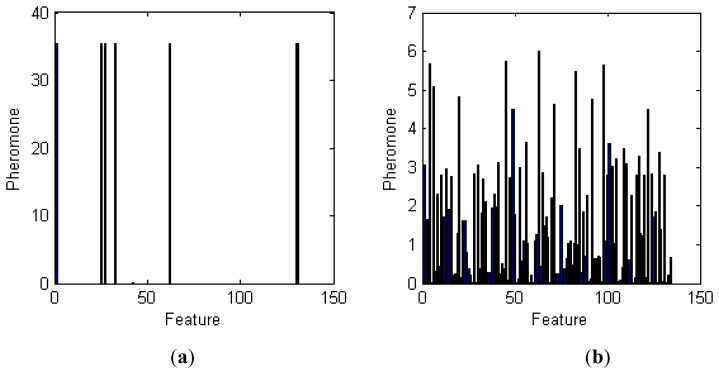
Pheromone trail of various features (x-axis denotes the index of selected features; y-axis denotes the pheromone trail of the given feature): (**a**) moving mode feature pheromone trails; and (**b**) stationary mode feature pheromone trails.

To understand the importance of each feature in the classification process, we used the method described in Section 3.6 to analyze the pheromone trail of those features. The results of this analysis are given in Figure 9.

As shown in Figure 9, the pheromone trails for moving mode features demonstrate good convergence. The indices of the best features are 1, 25, 33, 62, 130, 131, and 132. Features 1 to 129 are the results of DFFT and reflect the frequency-domain characteristics of acceleration. The frequencies of features 1, 25, 33, and 62 are 0, 4.6875, 6.2500, and 11.9141 (Hz), respectively. The indices of the minimum, maximum, mean, and standard deviation of velocity values for the time window are 130, 131, 132, and 133, respectively. The last one, 134, is the SampEn.

The convergence of feature pheromone trails for stationary mode is not nearly as obvious. The most important characteristic frequencies are 12.1094, 8.5938, 0.5859, 18.9453, 16.0156, 0.9766, and 3.7109 (Hz). Neither the speed feature nor the feature results of SampEn—which reflect the periodicity complexity and standard deviation of the velocity distribution—played a key role in the classification.

### 4.3. Example Loop Trajectory

To demonstrate the application of our classification method, we applied it to a simple loop trajectory. Although it was only an example, it was representative because it contained almost all transportation modes.

**Figure 10.**
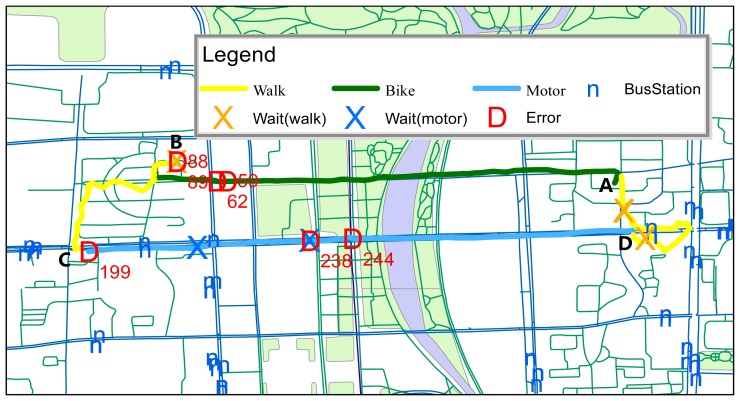
Classification of a travel loop involving multiple modes of transportation.

Our demonstration was the classification of a travel loop involving multiple modes of transportation. The travel loop is illustrated in Figure 10. A was the starting point. A subject left A on a bicycle and arrived at B. After parking the bicycle, the subject walked through a crossroads to reach C. The subject then boarded a bus at a station near C and rode it to D, where s/he walked through a crossroads and returned to starting point A. The wait mode sections while walking between A and B were produced by parking the bicycle. The wait mode sections while motoring between C and D were incurred by the bus waiting for traffic lights and stopping at stations. The wait mode sections while walking between sections D and A were produced by waiting for traffic lights and making another short pause.

There were 361 sections in the loop trajectory, including seven error sections. The total classification accuracy for the loop was 98.06%. The labels for the error sections were their section indices. Sections 59 and 62 were classified as motor mode; Sections 88 and 89 were classified under wait-while-walking Mode and stay mode, respectively, because the subject was parking his/her bike in those windows and did not flag the changed mode in time. Section 199 was classified under bike mode and Section 238 was wait-while-motoring; however the latter was identified as wait-while-walking because the subject walked onto a bus. Section 244 was identified as bike mode.

## 5. Conclusions

In this paper, we proposed a method of classifying user transportation modes based on motion and location data collected from smartphones. The method uses a refined classification model that not only identifies relevant transportation modes, but also elucidates the semantics of short pauses in physical movement to support more effective data mining. In our experimental evaluation, the proposed method achieved a relatively high classification accuracy of 96.31%. In our design and evaluation, we focused on minimizing requirements for hardware installation and user interaction. We chose a popular Android smartphone as our experimental platform and used sensor data collected only from the accelerometer and GPS receiver of this device.

We chose three groups of features: DFFT features to represent the frequency-domain characteristics of acceleration, a sample entropy feature to reflect the periodicity of acceleration, and the statistical features of velocity. In our proposed method, ACO is used to analyze and extract the most important features as inputs for SVM classifiers. This not only reduces feature dimensions, but also reveals the key factors in accurate classification, while further optimizing the system and elucidating the physical meaning of the extracted features.

The main focus of this study was on outdoors transportation modes that did not include subway, light rail, and similar urban transportation modes. In future work, we intend to focus on these transportation modes.

## Figures and Tables

**Table 1.** Location sources.

**Location**	**Features**
GPS	Highly accurate, directly provides velocity, only functions outdoors, quickly consumes battery power, and does not return the current location as quickly as some users may desire.
Cell towers	Works indoors and outdoors, quickly responds, uses less battery power than GPS, and is accurate up to 100 to 300 m, depending on the service provider. Cannot directly provide velocity.
WiFi	Works indoors and outdoors, quickly responds, uses less battery power than GPS, and is accurate up to 30 to 200 m, depending on the service provider. Cannot directly provide velocity.

**Table 2.** Sensor features.

Digital interface	SPI (4-wire, 3-wire), I^2^C, two interrupt pins VDDIO voltage range: 1.2 V to 3.6 V
Programmable functionality	Acceleration ranges: ±2 g/±4 g/±8 g/±16 g Low-pass filter bandwidths: 1 kHz – < 8 Hz
On-chip interrupt controller	Motion-triggered interrupt-signal generation for new data any motion (slope) detection tap sensing (single/double tap) orientation recognition flat detection low-g/high-g detection

**Table 3.** Segment numbers in various modes.

**Mode**	**Segment Number**
Walk	5570
Bike	4244
Motor	4177
Stay	2118

**Table 4.** Segment numbers after filtering.

**Mode**		**Segment Number**
Moving	Walk	5340
	Bike	4193
	Motor	3508

Stationary	Stay	2118
	Wait-while-walking	230
	Wait-while-biking	51
	Wait-while-motoring	669

**Table 5.** Computing time.

**Feature Number**	**Mode**	**Training (ms)**	**Predicting (ms)**
37	Moving	541.03	60.97
	Stationary	1776.87	210.62
134	Moving	2563.83	291.88
	Stationary	9927.25	740.41

**Table 6.** Average confusion matrices from moving mode.

	**Walk**	**Bike**	**Motor**
**Walk**	**0.9996**	0.0002	0.0002
**Bike**	0.0015	**0.9805**	0.0180
**Motor**	0.0006	0.0161	**0.9833**

**Table 7.** Average confusion matrices from stationary mode.

	**Stay**	**Wait** **(Walk)**	**Wait** **(Bike)**	**Wait** **(Motor)**
**Stay**	**0.9706**	0.0009	0.0066	0.0218
**Wait (Walk)**	0.0005	**0.9953**	0.0009	0.0033
**Wait (Bike)**	0.0024	0.0052	**0.9227**	0.0697
**Wait (Motor)**	0.0280	0.0043	0.1905	**0.7773**
